# *Ginkgo biloba* Extract EGb 761^®^ in Patients with Chronic Tinnitus: Treatment Effects and Effect Modifiers

**DOI:** 10.3390/jcm15010087

**Published:** 2025-12-23

**Authors:** Grażyna Lisowska, Irena Urban, Piotr Henryk Skarżyński, Sandra Schlaefke, Petra Brueggemann, Birgit Mazurek

**Affiliations:** 1 NZOZ Centrum Medyczne LIMED, Tylna 12, 42-600 Tarnowskie Góry, Poland; grazyna.lisowska@onet.pl; 2 Center of Hearing and Speech MEDICINUS, 05-830 Kajetany, Poland; i.urban@csim.pl (I.U.); p.skarzynski@csim.pl (P.H.S.); 3 Institute of Sensory Organs, 05-830 Kajetany, Poland; 4 Clinical Research, Dr. Willmar Schwabe GmbH & Co. KG, Willmar-Schwabe-Strasse 4, 76227 Karlsruhe, Germany; sandra.schlaefke@schwabe.de; 5 Tinnitus Center, Charité–Universitätsmedizin Berlin, 10117 Berlin, Germany

**Keywords:** EGb 761^®^, *Ginkgo biloba*, hearing impairment, anxiety, chronic tinnitus

## Abstract

**Background/Objectives**: An exploratory study was conducted to investigate the effect of *Ginkgo biloba* extract EGb 761^®^ in the management of chronic tinnitus, and whether comorbidities have an impact on the treatment outcome. **Methods**: The exploratory, uncontrolled, open-label study enrolled 170 patients (Full Analysis Set) with chronic tinnitus who took 120 mg EGb 761^®^ twice daily for 24 weeks. Outcomes were assessed using the Tinnitus Questionnaire, Tinnitus Handicap Inventory, and 11-Point Box Scales for loudness and annoyance. Comorbidities were recorded with audiometry, the Hospital Anxiety and Depression Scale, and the Perceived Stress Questionnaire. The effectiveness was further examined in responder analyses (at least 30% score reduction in 3 out of 4 outcomes) and in subgroups defined by baseline anxiety, hearing impairment, stress and depression. **Results**: At week 24, significant improvements were observed in all tinnitus-related outcomes compared to baseline (all *p* < 0.0001). In subgroup analyses, patients with high baseline anxiety or stress as well as those with normacusis improved more, whereas baseline depression had no influence. The overall response rate was 18.8%. **Conclusions**: The results of this exploratory study indicate that EGb 761^®^ improved complaints in patients with chronic tinnitus. The therapy appears to be particularly beneficial for patients with normal hearing and/or concomitant anxiety and/or stress. Trial registration: The study was registered at ISRCTN (ISRCTN83863387, registration date 14 October 2016).

## 1. Introduction

Tinnitus is the experience of sound without the presence of an external stimulus [1]. Individuals experiencing tinnitus report an unspecified acoustic sound such as ringing, pulsing, buzzing, or clicking [2]. Episodes of tinnitus may be acute (single-episode), intermittent, or chronic persistent (defined as lasting at least 3 months), which can last for many years [3,4]. Risk factors include hearing loss, ototoxic medication, head injury, and depression [5]. Due to variations in the definition of tinnitus and differences in studied populations, reported prevalence varies. In a 2022 meta-analysis of 83 publications, the pooled prevalence of any tinnitus was 14.4% among the general population worldwide, suggesting that tinnitus affects more than 740 million adults globally and is perceived as a distressing symptom by more than 120 million people [6]. This percentage corresponds with the findings of the European Tinnitus Survey (12 countries; n = 11,427), in which the pooled prevalence of any tinnitus was 14.7%. In the same survey, 16.5% of respondents in Poland reported experiencing any form of tinnitus, and 5.8% reported experiencing “bothersome” tinnitus [7]. A second epidemiological survey of adults in Poland (n = 10,349) found that tinnitus lasting more than 5 min was reported by 20.1% of the population and constant tinnitus was reported by 4.8% [8].

Tinnitus is a heterogeneous condition, with some people experiencing only mild discomfort and others having a significant negative impact on their cognitive abilities and emotional state [1,9]. It can lead to sleep disturbance and concentration problems [10] which can have a negative impact on quality of life [9]. It can also be exacerbated by audiovestibular symptoms such as hearing loss. In addition, the intensity and chronicity of tinnitus symptoms are perpetuated by comorbidities such as stress, anxiety, or depressive mood [11,12].

Therefore, treatment strategies for chronic tinnitus must be interdisciplinary. To date, there is no causal therapy of chronic tinnitus and no recommendation for drug therapy in national and international clinical practice guidelines. Cognitive behavioural therapy has the highest level of evidence for reducing tinnitus-related distress.

In Germany and other countries, medicinal products containing EGb 761^®^—the dried extract of *Ginkgo biloba* leaves—as an active substance are approved for the symptomatic or adjuvant treatment of patients with tinnitus of vascular or involutive origin. A mediation analysis indicated that EGb 761^®^ could be considered as an adjunctive treatment for tinnitus in elderly patients with dementia, with additional benefit in those with symptoms of depression or anxiety [13]. The present exploratory study investigated whether these comorbidities influence the effectiveness of EGb 761^®^ in chronic tinnitus.

## 2. Materials and Methods

### 2.1. Study Design

The primary objective of this multicentre, single-arm, open-label, exploratory clinical trial was to investigate whether comorbidities influence the treatment effect of EGb 761^®^ in terms of improvement and response rates. The secondary objective was to identify the patient groups that would derive the greatest benefit from EGb 761^®^ treatment. The publication manuscript was written in accordance with the STROBE checklist (Strengthening the Reporting of Observational studies in Epidemiology) [14].

The trial comprised a screening visit up to seven days prior to enrolment, a baseline visit, a control visit after 12 weeks, and a final study visit at week 24 ± 1. Telephone calls were scheduled for weeks 6 and 18 to assess adverse events and changes in concomitant medications. At the initial screening stage, patients underwent a diagnostic evaluation of tinnitus, encompassing an ear, nose, and throat examination. Subsequently, patients’ subjective hearing acuity was assessed with pure tone audiometry across a range of frequencies and volumes. At each study visit (baseline, week 12, and week 24), patients completed various questionnaires (see below).

### 2.2. Ethics Statement

The study was approved by the Ethics Committee at Silesian Medical Chamber in Katowice and conducted in accordance with the principles of Good Clinical Practice and the Declaration of Helsinki. Written informed consent was obtained from all participants. The study was prospectively registered at ISRCTN—The UK’s Clinical Study Registry (ISRCTN83863387, registration date 14 October 2016).

### 2.3. Participants

Patients were recruited in 12 investigational sites specialised to otorhinolaryngology or internal medicine in Poland between October 2016 and April 2017. Adults with unilateral or bilateral chronic tinnitus lasting for at least 3 months rated as grade 2 or 3 according to the Biesinger classification [15], with or without hearing loss, were eligible for inclusion. The criteria for the Biesinger classification are:

- Grade 2: tinnitus is mainly perceived in silence and is bothersome when the patient is under stress or strain.
- Grade 3: tinnitus causes permanent impairment of the patient’s personal and professional life; there is also presence of an emotional, cognitive, or physical disorder.

Patients with tinnitus due to Ménière’s disease, vestibular schwannoma, otosclerosis, acute or chronic otitis media or acute vestibular neuritis were excluded from the study. Those with an ongoing psychiatric illness or severe cardiac, circulatory, renal or hepatic disease were also ineligible to participate. Patients were also excluded if they had received cognitive behavioural therapy or tinnitus retraining within six months of the baseline visit, or had received any other treatment for tinnitus within two weeks of the baseline visit. Concomitant medications that were also prohibited included psychoactive drugs, systemic perfusion-enhancing drugs, anticholinergic drugs, cognition-enhancing drugs, and regular intake of anticoagulants.

### 2.4. Trial Medication

Patients were instructed to take one film-coated tablet containing 120 mg of the proprietary extract EGb 761^®^ (manufactured by Dr. Willmar Schwabe in Karlsruhe, Germany) twice daily for 24 weeks. EGb 761^®^ is a dry extract from the leaves of the *Ginkgo biloba* tree. One film-coated tablet is adjusted to contain 26.4–32.4 mg of ginkgo flavonoids and 6.48–7.92 mg of terpene lactones.

### 2.5. Outcomes

The main outcomes related to tinnitus were the 52-item version of the Tinnitus Questionnaire (TQ) [16], the Tinnitus Handicap Inventory (THI) [17], and the 11-point box scales for tinnitus loudness and tinnitus annoyance at week 12 and week 24 and their differences to baseline. The 11-point box scales range from 0 (indicating the absence of tinnitus, or a lack of annoyance, respectively) to 10 (representing the perception of extremely loud tinnitus or a high level of annoyance, respectively). It is a variant of the visual analogue scale, which is a valuable tool for assessing subjective experiences in individuals with chronic tinnitus [18]. Furthermore, the abbreviated TQ Mini score, comprising 12 items, was calculated to evaluate whether the abbreviated scale would be helpful in future research. It is easier to complete and has been shown to have similar qualities in terms of reliability and discriminatory power [19]. The Sheehan Disability Scale (SDS) is a self-report instrument designed to assess functional impairment in work, home, and family responsibilities on a 10-point numerical rating scale [20].

The assessment of anxiety and depression was conducted using the Hospital Anxiety and Depression Scale (HADS) [21]. A HADS anxiety subscore of 11 points or more was considered to indicate anxiety, while a score of less than 11 points was defined as normal or subsyndromal anxiety. A HADS depression subscore of 8 points or less was defined as normal, while scores of 8 points or more were defined as either subsyndromal or abnormal. Stress levels were measured with a validated Polish translation of the Perceived Stress Questionnaire (PSQ). The questionnaire comprises 30 items, designed to assess subjective stress experienced over the preceding four weeks. From the sum score, an index can be derived which ranges from 0 to 1 [22]. An index of <0.45 was defined as no stress, while an index ≥ 0.45 was considered as abnormal stress level.

Pure tone audiometry (air conduction) was used to determine the subject’s hearing thresholds. This method involves the application of a range of frequency-specific pure tones to which the patient responds. Patients were classified according to World Health Organisation criteria. They were considered mildly impaired if their hearing threshold (average of 500 Hz, 1000 Hz, 2000 Hz and 4000 Hz in the better ear) was 26–40 decibel hearing level (dB HL), moderate impairment 41–60 dB HL and severe impairment was 61–80 dB HL [23]. A binary variable was used for the analysis which distinguishes between no impairment (25 dB and less) and at least slight hearing impairment (26 dB and above).

For the responder analysis, a ‘slight’ response was defined as an improvement of at least 15% between baseline and week 24, while a ‘moderate’ response was defined as an improvement of at least 30%. This is reasonable, given that a minimally clinically important difference of 12 points is considered for the total TQ score change from baseline [24]. With a baseline score of almost 40 in the study population, this equates to an improvement of around 30%. The ‘overall response’ criterion was defined as an improvement of at least 30% in three of the four main treatment outcomes.

Safety and tolerability outcomes included the frequency and nature of adverse events (AEs) and the results of the physical examination, safety laboratory tests (haematology, clinical chemistry including liver function and blood coagulation parameters), and vital signs which were documented at screening and week 24.

### 2.6. Statistical Analysis

In view of the absence of clarity regarding the factors that influence the treatment effect of EGb 761^®^, no confirmatory hypothesis was formulated. Consequently, no formal sample size calculation was carried out. A number of 160 patients plus about 10% potential drop-outs, in total 175 patients seemed adequate for the analyses.

TQ total score, THI total score, tinnitus loudness and tinnitus annoyance as the main treatment outcomes were assessed through the implementation of an analysis of covariance (ANCOVA) model. In this model, the value observed at each follow-up visit served as the dependent variable, while the baseline value was utilised as a covariate. The TQ mini score and the SDS total score were analysed in the same way. ANCOVA models asume normality of residuals and homoscedasticity. Robustness properties of ANCOVA to arbitrary model misspecification have been proven by Wang et al. 2019 [25]. The results of the ANCOVA models are asymptotically valid even when the linear model is arbitrarily misspecified.

Any missing values at week 24 were replaced by the last observation carried forward (LOCF) method. For variables composed of single-item variables, the LOCF was applied on an item-by-item basis. Missing values at week 12 were not replaced by baseline values.

Continuous outcomes were described by the number of evaluable observations (N), arithmetic mean (mean) and standard deviation (SD). *p*-values of the ANCOVA models are presented.

Baseline characteristics and safety outcomes are presented by absolute frequencies and percentages for categorical variables and by means with standard deviations for continuous variables.

The treatment effects were examined in subgroups defined by baseline comorbidities, namely anxiety, hearing impairment, stress and depression. The differences in treatment effects between subgroups with and without hearing impairment, with and without depression or stress are presented by adjusted means, standard errors of adjusted means, and *p*-values for changes within subgroups as well as adjusted means differences, standard errors of adjusted mean differences, and *p*-values for the comparison of subgroups. The comparison of treatment effects between patients with and without anxiety was performed without adjustment for baseline scores.

Responder rates are presented as percentages based on the sample size of the full analysis set. Overall responders were characterized using stepwise regression analysis with the prognostic factors ‘age’, ‘sex’ and the baseline comorbidities hearing loss, anxiety (HADS anxiety, categorized), stress (PSQ, categorized) and depression (HADS depression, categorized). The significance levels for entry into the model and for staying in the model were set to alpha = 0.1. Odds ratios (ORs) with 95% confidence intervals and *p*-values were computed for factors included into the logistic regression model.

Statistical analyses were performed using SAS (Version 9.4). *p*-values < 0.05 are considered statistically significant. No adjustment has been made for multiplicity, on account of the exploratory nature of the analysis. *p*-values are to be regarded as standardised statistics which allow the comparison of treatment effects between different outcomes and subgroup analysis.

## 3. Results

### 3.1. Patient Characteristics

**Figure 1:**
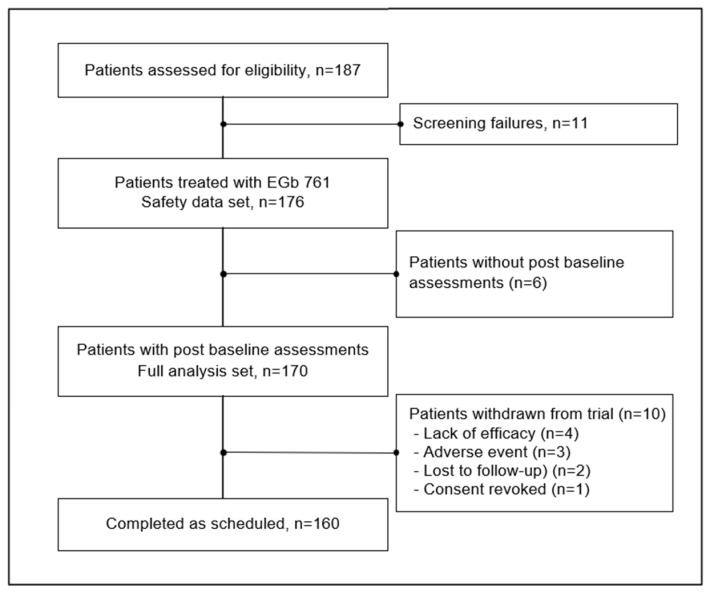
Disposition of subjects, analysis data sets.

In total, 187 patients were recruited of which eleven did not successfully complete the screening phase. The remaining 176 patients started treatment with EGb 761^®^ (safety set). A total of 16 (9.1%) patients terminated the trial prematurely during the active treatment phase. The study duration was between 9 and 210 days with a mean of 163.4 days and a standard deviation of 33.5 days (safety set, n = 176). Six patients were excluded from efficacy analyses since no measurements of the efficacy outcomes after baseline were available. The full analysis set thus included 170 patients (Figure 1). For 6 of these patients no efficacy outcomes in week 12 were available and for 5 patients no efficacy assessments took place at the end of the treatment period (week 24). Missing values in week 24 were replaced by the last observation carried forward to analyse all patients taking their individual treatment periods into account. Unless otherwise stated, all results are presented for the full analysis set. Baseline characteristics of patients are shown in Table 1.

### 3.2. Effectiveness Outcomes

Of the patients in the FAS, 65.3% reported taking at least one additional medication alongside EGb 761^®^, primarily antibiotics, cardiovascular drugs, sex hormones, urological drugs and levothyroxine.

**Figure 2:**
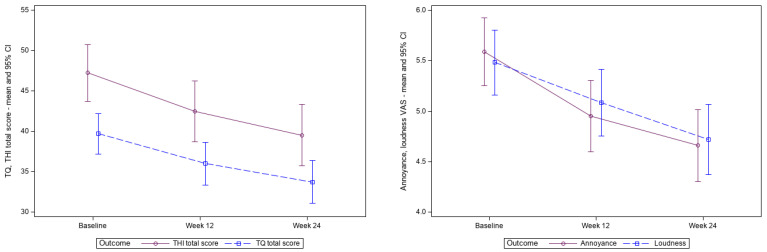
TQ total score, THI total score, tinnitus loudness and annoyance during the trial (mean and 95% confidence interval). Abbreviations: THI, Tinnitus handicap inventory; TQ, Tinnitus questionnaire.

The mean values of the variables employed to assess treatment effects—the TQ total score, TQ mini, THI total score, 11-Point Box Scales for tinnitus loudness and tinnitus annoyance—exhibited a continuous decrease until weeks 12 and 24, in comparison to the baseline values (Table 2). The difference was found to be significant at both time points (range *p* < 0.0001 to *p* = 0.0011, Table 2). This pattern demonstrated a continuous improvement in symptoms (Figure 2).

The SDS total score decreased from mean 8.3 points (FAS) at baseline by −1.0 ± 4.8 (SD) points until week 12 and by −1.9 ± 5.7 (SD) points until week 24, which was statistically significant (*p* ≤ 0.0158 for both visits).

### 3.3. Subgroup Analyses by Comorbidities

**Figure 3:**
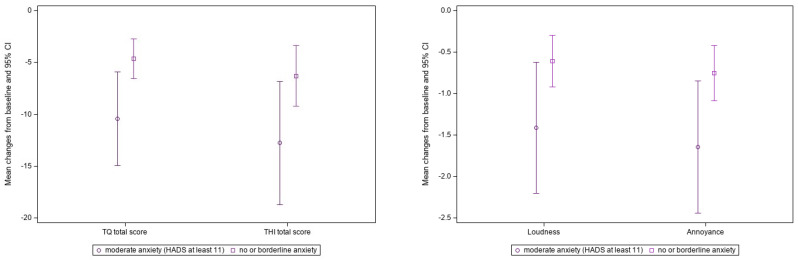
Treatment effects in patients without/subsyndromal anxiety versus those with anxiety. Abbreviations: HADS, Hospital Anxiety and Depression Scale; THI, Tinnitus handicap inventory; TQ, Tinnitus questionnaire. (*p* = 0.0211/0.0.0533/0.0606/0.0408 for TQ total score/THI total score/tinnitus loudness/annoyance comparing patients with anxiety and patients with subsyndromal or no anxiety).

In patients with HADS anxiety scores ≥ 11 points, and in patients with subsyndromal or no anxiety, there was a significant improvement in TQ, TQ mini, THI, tinnitus loudness and tinnitus annoyance scores between baseline and week 24 (*p* < 0.001 for all endpoints in both subgroups) (Table 3). Furthermore, the group with anxiety exhibited a significantly greater improvement in TQ, TQ mini and tinnitus annoyance compared to the group without or with subsyndromal anxiety (Table 3, Figure 3).

At baseline, 42 patients had mild hearing loss, 8 had moderate hearing loss and 2 had severe hearing loss. The remaining 118 patients had no hearing impairment (Table 1). Changes in efficacy outcomes are more pronounced in the subgroup of patients without hearing impairment compared to patients with at least mild hearing loss. The analysis revealed no statistically significant differences between the subgroups in terms of change in TQ total score, THI total score, tinnitus loudness and annoyance (*p* > 0.05). Regarding the 12 items of the TQ mini (short version of the TQ), there is a statistically significant difference between the subgroups (*p* = 0.0486).

The effect of stress was examined using the PSQ index threshold of 0.45 (Table S1, Figure S1 in the Supplemental File). In the subgroups of patients with and without stress, TQ, TQ mini, THI, tinnitus loudness, and tinnitus annoyance scores decreased significantly (*p* ≤ 0.001 for all endpoints in both groups) by week 24. A statistically significant difference was observed in the reduction of tinnitus loudness (*p* = 0.0478) and tinnitus annoyance (*p* = 0.0403) at week 24 between the group of patients with a PSQ stress index of ≥0.45 and those with a PSQ stress index of <0.45, indicating that patients with higher stress levels showed greater improvement.

In patients with and without abnormal HADS depression scores at baseline (Table S2 in Supplemental File), TQ, THI, tinnitus loudness, and tinnitus annoyance scores improved significantly by week 24 (*p* ≤ 0.001 for all endpoints in both groups). There were no significant differences between patients with and without depression (*p* > 0.05 for all endpoints); patients improved to a similar extent, regardless of baseline depression.

### 3.4. Responder Analysis

**Figure 4:**
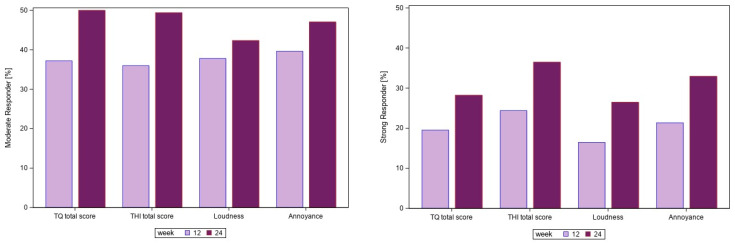
Responder analysis—percentage of patients with a slight or moderate response at weeks 12 and 24 (N = 170). Abbreviations: THI, Tinnitus handicap inventory; TQ, Tinnitus questionnaire.

At week 24, the proportion of patients with at least 30% improvement ranged from 26.5% for tinnitus loudness to 36.5% for THI. Nearly half of the patients (42.4% for tinnitus loudness, 50% for TQ) exhibited an improvement from baseline between 15% and 30%. Approximately 50% of the patients did not experience a meaningful improvement. The results of the responder analysis are presented in Figure 4.

An improvement of at least 30% between baseline and week 24 in at least 3 of the 4 main efficacy outcomes (overall response) was observed for 32 of 170 patients (18.8%). The most significant factors contributing to treatment response were identified as no hearing loss (odds ratio [OR] of 4.42, 95% CI [1.42; 13.70], *p* = 0.0102) or anxiety (HADS anxiety ≥ 11 points, OR of 2.62, 95% CI [1.05; 6.54], *p* = 0.0391). The other factors which might be correlated with anxiety or hearing impairment were not selected in the stepwise logistic regression analysis. In total 20 patients without hearing impairment suffered from anxiety. Eight of these patients (40.0%) were overall responders (improvement of at least 30% between baseline and week 24 in at least 3 of the 4 main efficacy outcomes).

### 3.5. Safety

The treatment of chronic tinnitus with EGb 761^®^ over 24 weeks was safe and well tolerated. The mean exposure was 159 ± 35.5 days (safety analysis set, n = 176). The mean drug compliance was 97.2 ± 9.3%. During treatment and the following 2 days after the last dose of EGb 761^®^, 37 patients (21.0%) experienced a total of 52 adverse events (AEs). The majority was classified as mild or moderate in intensity.

The most common AEs (independently of the associated causality assessment) were upper respiratory tract infection (n = 6), pharyngitis (n = 5), headache (n = 3), palpitations (n = 2), bronchitis (n = 2), and influenza (n = 2). One patient experienced a serious AE (acute renal colic due to urolithiasis) that was considered as unrelated to EGb 761^®^.

A total of 19 AEs occurred in 15 patients for which a causal relationship with EGb 761^®^ could not be excluded and the causal relationship for the vast majority was assessed as unlikely. The most common AEs were nervous system disorders (n = 4), gastrointestinal disorders and general disorders (n = 3 each). All ADRs were mild or moderate in intensity and resolved by the end of the study or during follow-up. and the causal relationship for the vast majority was assessed as unlikely.

No statistically significant alterations in mean, minimum, or maximum laboratory parameters were observed between the screening visit and the week 24 visit. Heart rate and mean systolic and diastolic blood pressures were similar before and after treatment with EGb 761^®^. No changes were observed in the physical examinations throughout the study, except for one patient who had pain and swelling of the thyroid gland at week 24. The patient was diagnosed with subacute thyroiditis, which was documented to be unrelated to EGb 761^®^.

## 4. Discussion

The present trial showed significant improvements in the main tinnitus-related outcomes (TQ total score, 12-item TQ mini, THI total score, 11-Point Box Scales for tinnitus loudness and tinnitus annoyance) after 24 weeks of treatment with EGb 761^®^ in patients with chronic tinnitus. Noteworthy, at baseline more than 80% of patients reported a tinnitus duration of more than one year. These results indicate the effectiveness of EGb 761^®^ in tinnitus management, regardless of the duration of the condition.

Score reductions in tinnitus-relevant measures in this study demonstrated improvements in tinnitus loudness/severity and overall severity in about half of the patients. However, statistically significant effects are not necessarily clinically relevant [26,27]. Positive effects should therefore be confirmed in a randomized controlled study. The good safety and tolerability of EGb 761^®^ demonstrated in this study are consistent with the results of previous studies and reviews [28].

A post-hoc analysis of pooled data (n = 594) from older adults with tinnitus and mild to moderate dementia has demonstrated a close link between tinnitus, depression and anxiety [13]. The analysis suggests that anxiety and depression may influence tinnitus severity. In the subgroup analysis of comorbidities, patients with baseline anxiety, normacusis and elevated stress level showed a significantly better response in the TQ mini. The reduction in the THI of 12.8 ± 17.0 (mean ± SD) in patients with baseline anxiety, of 8.6 ± 1.6 (adjusted mean ± SE) in patients with normal hearing and of 8.2 ± 2.3 (adjusted mean ± SE) in stressed individuals was in the range of the minimal clinically important difference of 7.8 to 12 points [29]. In this study, baseline depression had no impact on the treatment outcomes with EGb 761^®^ whereas a retrospective cohort study indicated that depression severity is a predictor for treatment (other than EGb 761^®^) success [30].

An improvement of at least 30% in at least 3 of the 4 main efficacy outcomes defined as overall response was observed for 18.8% of patients. For patients without hearing loss and high anxiety levels an overall response rate of 40% was observed. These patients benefited particularly from treatment with EGb 761^®^.

The mechanism of action underlying the effect of EGb 761^®^ on chronic tinnitus is not fully understood [31]. It is postulated that the enhancing the cochlear blood flow, protection against ototoxic stimuli, inhibition of apoptosis and impedance of age-associated degenerative processes in the inner ear, as well as effects on neurotransmitters, are pharmacologic actions. In a rat stress model, EGb 761^®^ normalises dopamine and norepinephrine levels in the brain, as well as plasma stress hormone levels. Enhancement of the dopaminergic system has been reported in both animals and isolated cochlear cells. Furthermore, repeated administration of EGb 761^®^ modulates cholinergic neurotransmission, likely via the elevation of muscarinic acetylcholine receptors in the hippocampus [31]. Results from a placebo-controlled clinical trial of EGb 761^®^ in elderlies with subjective memory impairment also suggested enhancement of prefrontal dopaminergic activity [32]. Dysregulation of neurotransmitters, including dopamine, is involved in the pathogenesis of tinnitus, as well as mood disorders such as anxiety, stress and depression [32]. Consistent with these findings, dopaminergic drugs such as pramipexole, sulpiride and melatonin have been reported to improve subjective tinnitus [33,34]. However, these data require further evaluation and have not yet been incorporated into treatment guidelines.

In this study, treatment response was seen in patients with anxiety and those without hearing loss. In the latter, decreased inhibition due to partial cochlear neural degeneration is thought to trigger hyperactivity in the central nervous system as a possible pathogenetic mechanism [35]. The mechanism of action of EGb 761^®^ may involve a global inhibitory process that helps to counteract tinnitus. In an animal model of noise-induced tinnitus, three weeks of treatment with EGb 761^®^ resulted in an improvement in behavioural signs of tinnitus that was accompanied by a persistent recovery of auditory thresholds back to pre-trauma conditions. Auditory brainstem response wave analyses indicated an increase in response to low stimulus intensities and a decrease to high intensity stimulation in EGb 761^®^ treated animals [36].

Other studies investigating the efficacy of *Ginkgo biloba* extracts for the treatment of tinnitus have yielded mixed results. In a randomised controlled trial, EGb 761^®^ and pentoxifylline were similarly effective in reducing tinnitus loudness and annoyance [37]. An older systematic literature review of eight placebo-controlled trials concluded that EGb 761^®^ was significantly more effective than placebo in the treatment of tinnitus [38]. A review of 15 studies using different *Ginkgo biloba* products for the treatment of tinnitus found conflicting results [39], and a Cochrane review of 2022 concluded that there is uncertainty about the benefits and harms of *Ginkgo biloba* for the treatment of tinnitus when compared to placebo [40]. However, the efficacy of a plant extract depends on its composition, which is influenced by the manufacturing process, the bioavailability of its active compounds, and its dosage [41]. Products made from the same plant species using different production processes may not be bioequivalent and thus findings from studies of one specific extract should not be generalised to other products and vice versa [41].

There are several limitations to interpreting the trial results. Firstly, the open-label, single-arm design is a significant limitation. Studies without randomisation and control groups tend to be subject to inherent biases, making it difficult to assess the effect of treatment. Therefore, the reported improvements cannot be confidently attributed to EGb 761^®^. Furthermore, chronic tinnitus is characterised by fluctuations, so regression to the mean is likely. Expectation bias also plays a substantial role in tinnitus trials and may have influenced the results, particularly given the subjective nature of the outcomes. The design was chosen as it reflects the treatment situation in everyday clinical practice with the aim of generating hypotheses and descriptive statistical analysis. Since there is limited information on whether causes, risk factors, chronicity, characteristics of tinnitus and associated features influence the treatment effect of EGb 761^®^, this approach is reasonable. Consistent results from analyses of different tinnitus-related outcomes support the validity and integrity of the data. Nevertheless, future studies are needed to further investigate the hypotheses generated by this study.

## 5. Conclusions

Treatment with EGb 761^®^ significantly improved all tinnitus-related outcomes for nearly half of the patients with chronic tinnitus. The enhancement of a central inhibitory mechanism may underlie the clinical effect. Patients without hearing loss and high levels of anxiety demonstrated the most favourable treatment response. Therefore, EGb 761^®^ treatment for chronic tinnitus could be an interesting option for these patients.

## Figures and Tables

**Table 1:** Patient baseline characteristics.

Parameter	Total (N = 170)
**Age (years)**	
Mean ± SD	51.6 ± 13.0
Min, max	21, 82
**Gender**	
Female	62 (36.5%)
Male	108 (63.5%)
**Tinnitus characteristics, n (%) ^†^**	
Bilateral	87 (51.2%)
Permanent	149 (87.6%)
Duration > 1 year	138 (81.2%)
**Comorbidities**	
**Patients with hearing impairment, n (%)**	
No impairment	118 (69.4%)
At least mild impairment	52 (30.6%)
**HADS anxiety, n (%)**	
Normal or subsyndromal (0–10 points)	135 (79.4%)
Abnormal (11–21 points)	34 (20.0%)
**HADS anxiety,** mean ± SD	7.7 ± 3.9
**HADS depression, n (%)**	
Normal (0–7 points)	111 (65.3%)
Subsyndromal or abnormal (8–21 points)	58 (34.1%)
**HADS depression,** mean ± SD	5.5 ± 4.0
**PSQ (stress index), n (%)**	
Normal (<0.45)	112 (65.9%)
Abnormal (≥0.45)	58 (34.1%)
**PSQ stress index**, mean ± SD	0.389 ± 0.159

^†^ Several characteristics and conditions can occur at the same time. Abbreviations: Min, Minimum; Max, Maximum; PSQ, Perceived Stress Questionnaire; SD, Standard deviation; HADS, Hospital Anxiety and Depression Scale.

**Table 2:** Overall treatment effects—baseline scores and improvements at weeks 12 and 24.

Outcomes	Baseline (N = 170)	Improvement, Week 12 (N = 164) *		Improvement, Week 24 (N = 170)	
	Mean (±SD)	Mean (±SD)	*p*-value	Mean (±SD)	*p*-value
**TQ (points)**	39.7 (±16.5)	−3.8 (±9.7)	*p* < 0.0001	−6.0 (±11.9)	*p* < 0.0001
**TQ mini (12 items)**	12.4 (±5.3)	−1.3 (±3.5)	*p* < 0.0001	−2.1 (±4.2)	*p* < 0.0001
**THI (points)**	47.2 (±23.4)	−4.7 (±14.7)	*p* = 0.0001	−7.7 (±17.3)	*p* < 0.0001
**Loudness (points)**	5.5 (±2.1)	−0.4 (±1.7)	*p* = 0.0011	−0.8 (±1.9)	*p* < 0.0001
**Annoyance (points)**	5.6 (±2.2)	−0.7 (±1.9)	*p* < 0.0001	−0.9 (±2.0)	*p* < 0.0001

* 6 patients without efficacy assessment in week 12. Abbreviations: SD, standard deviation; THI, Tinnitus handicap inventory; TQ, Tinnitus questionnaire.

**Table 3:** Analysis of the influence of baseline anxiety on EGb 761^®^ treatment effects.

Outcomes	Patients with Anxiety, HADS ≥ 11 (n = 34)		Patients with Subsyndromal or No Anxiety, HADS < 11 (n = 135)		Group Difference ^†^
	Baseline Mean ± SD	Improvement, Week 24 Mean ± SD, *p*-Value *	Baseline Mean ± SD	Improvement, Week 24 Mean ± SD, *p*-Value *	*p*-Value ^†^
**TQ (points)**	51.0 ± 14.6	−10.4 ± 13.0, *p* < 0.0001	35.3 ± 14.8	−4.6 ± 11.2, *p* = 0.0005	0.0211
**TQ mini (points)**	16.1 ± 4.4	−3.5 ± 4.3, *p* < 0.0001	11.4 ± 5.1	−1.6 ± 4.1, *p* < 0.0001	0.0263
**THI (points)**	66.5 ± 19.4	−12.8 ± 17.0, *p* = 0.0001	42.3 ± 22.2	−6.3 ± 17.3, *p* < 0.0001	0.0536
**Loudness (points)**	6.1 ± 2.1	−1.4 ± 2.3, *p* = 0.0009	5.2 ± 2.0	−0.6 ± 1.8, *p* = 0.0002	0.0606
**Annoyance (points)**	6.6 ± 2.1	−1.6 ± 2.3, *p* = 0.0002	5.4 ± 2.1	−0.8 ± 1.9, *p* < 0.0001	0.0418

^†^ Comparison of changes from baseline; * Changes from baseline within groups; Abbreviations: HADS, Hospital Anxiety and Depression Scale; SD, standard deviation; THI, Tinnitus handicap inventory; TQ, Tinnitus questionnaire.

## Data Availability

The datasets presented in this article are not readily available because raw data cannot be shared both due to ethical reasons and to data protection laws. To the extent permitted by law, the study data required for validation purposes have been disclosed on corresponding databases. Reasonable requests to access the datasets should be directed to the corresponding author.

## Supplementary Materials

The following supporting information can be downloaded at: https://www.mdpi.com/article/10.3390/jcm15010087/s1, Figure S1: Treatment effects in patients with normal versus those with elevated stress levels (N = 170); Table S1: Analysis of the influence of baseline stress on EGb 761 treatment effects; Table S2: Analysis of the influence of baseline depression on EGb 761 treatment effects.
