# Supplementary material for: Ginkgo biloba Extract EGb 761® in Patients with Chronic Tinnitus: Treatment Effects and Effect Modifiers
Source: J Clin Med. 2025 Dec 23;15(1):87. doi: 10.3390/jcm15010087 (PMC12787070; doi:10.3390/jcm15010087)
Supplement: Supplementary file 1 [file jcm-15-00087-s001.zip › jcm-4016766-supplementary.pdf]

Supplementary materials

Table S1: Analysis of the influence of baseline stress on EGb 761 treatment effects.

Outcomes	PSQ stress index score ≥ 0.45 (n=58)		Normal, PSQ < 0.45 (n=112)		Group difference†
	Baseline Mean ± SD	Improvement, week 24 Adjusted mean ± SEM, p-value*	Baseline Mean ± SD	Improvement, week 24 Adjusted mean ± SEM, p-value*	p-value†
TQ (points)	43.3 ± 16.0	-6.5 ± 1.5, p<0.0001	37.8 ± 16.5	-5.7 ± 1.1, p<0.0001	p=0.6669
TQ mini (points)	14.1 ± 5.0	-2.4 ± 0.5, p<0.0001	11.5 ± 5.3	-1.9 ± 0.4, p<0.0001	p=0.4729
THI (points)	55.3 ± 22.9	-8.2 ± 2.3, p=0.0003	43.0 ± 22.6	-7.4 ± 1.6, p<0.0001	p=0.7584
Loudness (points)	5.7 ± 2.2	-1.1 ± 0.2, p<0.0001	5.4 ± 2.1	-0.6 ± 0.2, p=0.0010	p=0.0478
Annoyance (points)	5.9 ± 2.3	-1.3 ± 0.2, p<0.0001	5.4 ± 2.2	-0.7 ± 0.2, p<0.0001	p=0.0403

† Comparison of changes from baseline, * Changes from baseline within groups;
Abbreviations: PSQ, Perceived Stress Questionnaire; SEM, standard error of mean; SD, standard deviation; THI, Tinnitus handicap inventory; TQ, Tinnitus questionnaire.

Table S2: Analysis of the influence of baseline depression on EGb 761 treatment effects.

Outcomes	Depression HADS ≥ 8 (n=58)		No Depression HADS < 8 (n=111)		Group differences†
	Baseline Mean ± SD	Improvement, week 24 Adjusted mean ± SEM, p-value*	Baseline Mean ± SD	Improvement, week 24 Adjusted mean ± SEM, p-value*	p-value†
TQ (points)	48.1 ± 15.1	-5.4 ± 1.6, p=0.0008	35.3 ± 15.6	-6.3 ± 1.1, p<0.0001	0.6375
TQ mini (points)	15.0 ± 4.7	-1.9 ± 0.5, p=0.0009	11.0 ± 5.1	-2.2 ± 3.9, p<0.0001	0.6095
THI (points)	59.4 ± 21.1	-7.7 ± 2.3, p=0.0010	40.9 ± 22.1	-7.9 ± 1.6, p<0.0001	0.9662
Loudness (VAS)	5.9 ± 1.7	-0.8 ± 0.2, p=0.0010	5.3 ± 2.3	-0.7 ± 0.2, p<0.0001	0.8313
Annoyance (VAS)	6.1 ± 1.9	-1.0 ± 0.3, p=0.0001	5.3 ± 2.3	-0.9 ± 0.2, p<0.0001	0.7285

† Comparison of changes from baseline, * Changes from baseline within groups;
Abbreviations: HADS, Hospital Anxiety and Depression Scale; SEM, standard error of mean; SD, standard deviation; THI, Tinnitus handicap inventory; TQ, Tinnitus questionnaire.

Figure S1: Treatment effects in patients with normal versus those with elevated stress levels (N=170).

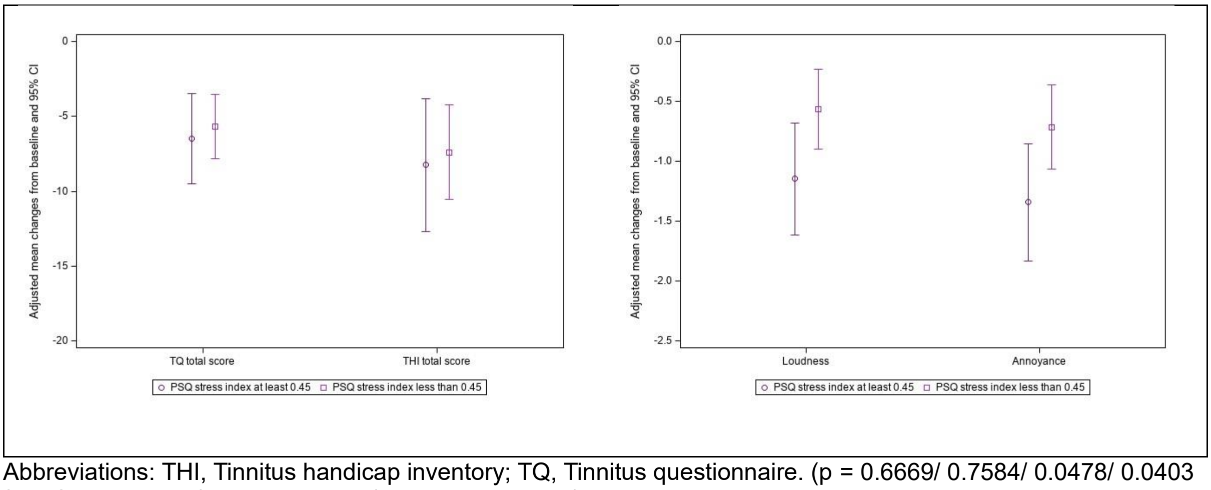

Abbreviations: THI, Tinnitus handicap inventory; TQ, Tinnitus questionnaire. (p = 0.6669/ 0.7584/ 0.0478/ 0.0403 for TQ total score / THI total score / tinnitus loudness / annoyance comparing patients with elevated stress levels and patients with normal stress levels)
